# MRI comparison of injury mechanism and anatomical factors between sexes in non-contact anterior cruciate ligament injuries

**DOI:** 10.1371/journal.pone.0219586

**Published:** 2019-08-01

**Authors:** Won Rak Choi, Jae-Hyuk Yang, Soo-Young Jeong, Jin Kyu Lee

**Affiliations:** 1 Department of Orthopaedic Surgery, Hanyang University Seoul Hospital, Seongdong-gu, Seoul, Korea; 2 Department of Orthopaedic Surgery, Hanyang University Guri Hospital, Guri, Gyeonggi-do, Korea; Northwestern University Feinberg School of Medicine, UNITED STATES

## Abstract

Non-contact anterior cruciate ligament (ACL) rupture is mostly caused by a pivot shift mechanism including valgus collapse and internal tibial rotation. In female athletes, the incidence of ACL rupture has been reported to be significantly higher than in their male counterparts. However, to date, there have been limited reports and controversy regarding sex differences underlying injury mechanisms of ACL and severity of injury. In this study, we hypothesized that 1) in patients with non-contact ACL rupture, the incidence and severity of pivot shift injury, which are determined by injury pattern on MRI, would be significantly higher in females, and 2) anatomical factors associated with pivot shift injury would be significantly associated with female sex. A total of 148 primary ACL ruptures (145 patients) caused by non-contact injury mechanisms were included in this study. Among them, 41 knees (41 patients) were female and 107 knees (104 patients) were male. The status of the osseous lesions, lateral and medial tibial slope, depth of the medial tibial plateau, collateral ligaments, and menisci were assessed by MRI and compared between sexes. The severity of osseous lesions at the lateral tibial plateau, lateral femoral condyle, medial tibial plateau, and medial femoral condyle were comparable between sexes. There were no significant differences between sexes in the location of tibial contusions (p = 0.21), femoral contusions (p = 0.23), or meniscus tears (p = 0.189). Lateral tibial slope was found to be significantly larger in females (8.95° vs. 6.82°; p<0.0001; odds ratio = 1.464), and medial tibial depth was significantly shallower in females (1.80mm vs. 2.41mm; p<0.0001; odds ratio = 0.145). In conclusion, females showed greater lateral tibial slope and shallower medial tibial depth compared to males, however it did not affect the sex differences in injury pattern.

## Introduction

Non-contact anterior cruciate ligament (ACL) rupture is mostly caused by a pivot shift mechanism including valgus collapse and internal tibial rotation.[1–6] In female athletes, the incidence of ACL rupture has been estimated to be >2- to 4-fold higher than in their male counterparts[7–9] with multifactorial causes related to anatomy, neuromuscular control, hormone levels, and biomechanical differences between sexes.[10–17]

Suggested anatomical factors typical in females include a narrow intercondylar notch, large Q-angle, small ACL size including length and cross-sectional area, increased posterior slope especially in lateral tibial plateau, and shallow medial tibial plateau.[10–16] Video analysis studies have shown that females tend to land with greater hip adduction, knee valgus, and lower knee flexion angles than males.[1, 11, 18–20] Landing at a low flexion angle leads to a large anterior translational force generated by an extensor mechanism and increased strain to the ACL.[21] Thus, valgus collapse may be more common in females and the mechanism of injury between females and males could be different.

There have been a few studies investigating differences in injury patterns between sexes via MRI. Fayad et al. reported a higher incidence of posterolateral tibial bone contusions in females and more severe lateral femoral condyle contusions and soft tissue injuries in males.[22] On the other hand, Wittstein et al. reported no differences in injury patterns or the severity of bone contusions and meniscus tears.[23] However, to date, limited information is available regarding whether sex differences exist in terms of ACL injury mechanisms and severity.

In this study, we hypothesized that 1) in patients with non-contact ACL rupture, the incidence and severity of pivot shift injury, which are determined by injury pattern on MRI, would be significantly higher in females, and 2) anatomical factors associated with pivot shift injury (e.g., large tibial slope and shallow depth of medial tibial plateau) would be significantly associated with female sex.

## Materials and methods

### Patients

The present study had no potential conflicts of interest. The Review Board on Human Subjects Research and Ethics Committees approved the study protocol. All patients provided written informed consent.

Data from this study cannot be shared publicly because it’s from human research participants. Data are available from the Review Board on Human Subjects Research and Ethics Committees of Hanyang university hospital for researchers who meet the criteria for access to confidential data.

Patients who underwent primary arthroscopic ACL reconstruction after confirmation of total rupture of ACL at Hanyang University Hospital between January 2012 and June 2018 were assessed for study eligibility. A retrospective review of medical records and radiographs was performed. After excluding patients with MRI taken more than 6 weeks after injury (10 patients), poor quality of MRI (5 patients), a history of ipsilateral knee surgery or injury (3 patients), unclear injury mechanism (i.e., could not confirm a non-contact mechanism) (21 patients), and knee dislocation (2 patients), a total of 148 ACL ruptures (145 patients) caused by non-contact injury mechanisms were included in this study. Among them, 41 knees (41 patients) were female and 107 knees (104 patients) were male.

Sports activity level at the time of injury was graded from 0 to 3 based on the Cincinnati knee rating system.[24, 25] Grade 0 was defined as activity of daily living (e.g., slip down at walking, twisted at climbing/going down the stairs). Grade 1 was defined as light sport with no running, twisting or jumping (e.g., golf, bowling, hiking, cycling, swimming). Grade 2 was defined as moderate sport with jumping, landing, running, twisting or turning (e.g., tennis, racquetball, handball, ice hockey, field hockey, skiing, wrestling). Grade 3 was defined as strenuous sport with hard twisting, cutting or pivoting (e.g., soccer, football, basketball, volleyball, gymnastics).

### MRI evaluation

Meniscal tear was diagnosed on the basis of MRI as suggested by De Smet et al.[26] but was not classified in the tear patterns. Collateral ligament injuries were graded according to the recommendations of Schweitzer et al.[27] and only grade 2 and 3 tears were counted for the purposes of this study. Posterior cruciate ligament tears were also evaluated and only grade 2 and 3 injuries were regarded as tears.

Osseous lesions including bone contusions and subchondral fractures were evaluated by the intensity and extent of abnormal edema-like marrow signal on the femoral condyles, tibial plateau, and fibular head according to the grading system described by Brittberg et al.[28]

**Fig 1:**
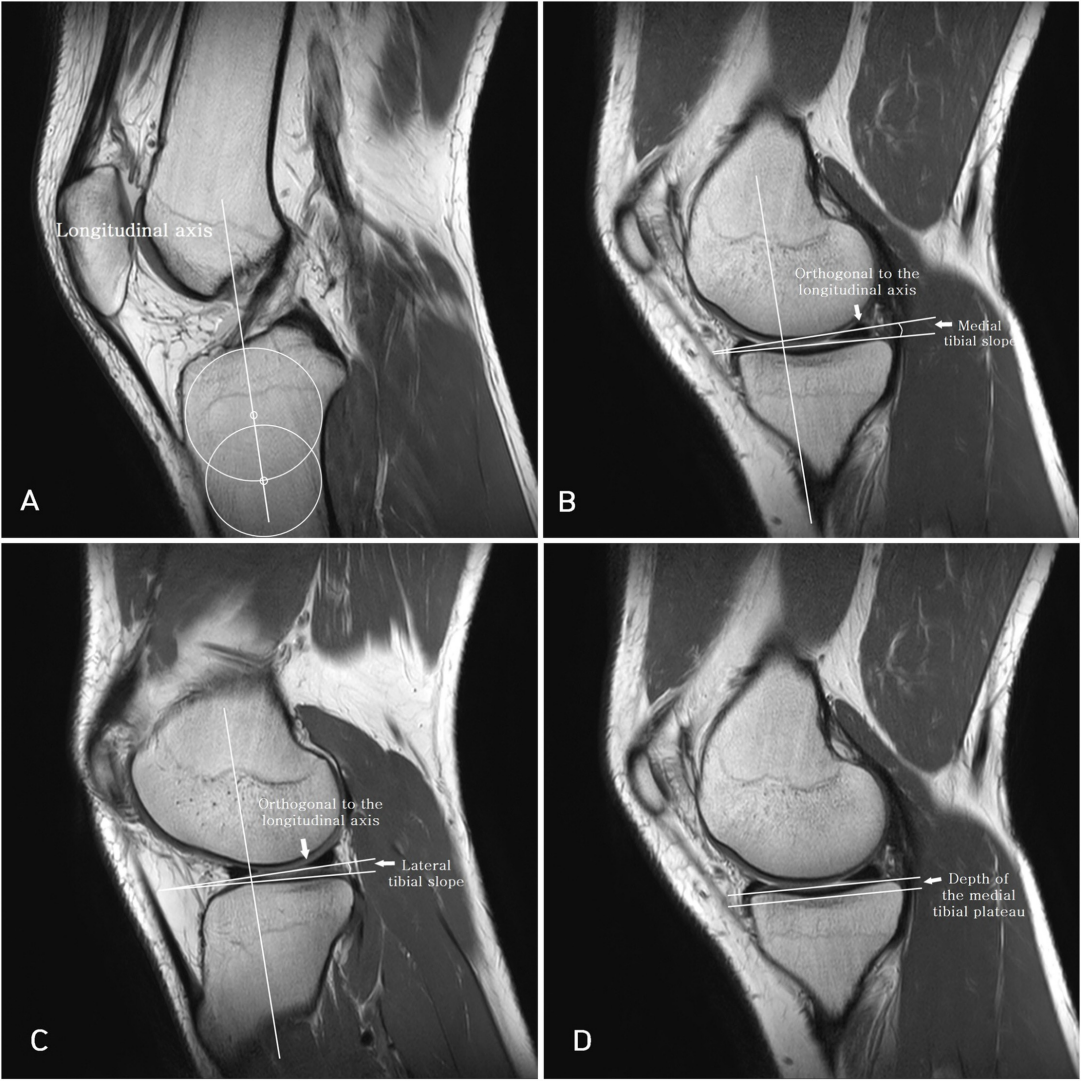
(A) shows the longitudinal axis of the tibia which is an extended line connecting 2 midpoints of the two circles. (B) shows the MTS at midsagittal plane of the medial tibial plateau with preserved longitudinal axis. (C) shows the LTS at midsagittal plane of the lateral tibial plateau with preserved longitudinal axis. (D) shows MTD at midsagittal plane of the medial tibial plateau. The perpendicular distance between the 2 lines was measured.

Medial tibial slope (MTS), lateral tibial slope (LTS), and depth of the medial tibial plateau (MTD) were measured by a method proposed by Hudek et al. (Fig 1)[29, 30] First, the longitudinal axis of the tibia in the sagittal plane was determined as an extended line connecting 2 midpoints of the two circles drawn within the proximal tibia. The proximal circle was fit within the proximal, anterior, and posterior cortical borders. The distal circle had to touch the anterior and posterior cortical borders and the center of the distal circle was positioned on the circumference of the proximal circle.

MTS and LTS were then measured as the angle between a line perpendicular to the longitudinal axis and a line joining peak points on the anterior and posterior aspects of the plateau at the sagittal plane, which was closest to the center of the articular surface. The MTD was measured on the same sagittal plane as the MTS was measured. A line connecting the anterior and posterior crests of the tibial plateau was drawn. A line parallel to this line was then drawn tangent to the lowest point of the concavity representing the lowest boundary of the subchondral bone. The perpendicular distance between the 2 lines was defined as the MTD.

### Statistical analysis

Student’s t-test was used to test whether the differences between the groups were significant. Other characteristics were compared by the Chi-square test and Fisher’s exact test. Multivariate logistic regression analysis was carried out with variables with p<0.05 in the univariate analysis (MTS, LTS, and MTD). For evaluation of inter- and intra-observer reliability, all MR images were reviewed by two independent orthopedic surgeons, and re-evaluated after 2 weeks by each assessor. Cohen’s kappa (κ) and intraclass correlation coefficient (ICC) values were calculated, and κ > 0.81 indicated a high association. Power analysis was conducted and on the basis of the results of Kim et al.[31], the calculation showed that 20 cases would be needed in each group to reach an alpha value of 0.05 and a power of 90% to detect a difference between sexes in valgus injury rate. Data analyses were done using SPSS v20.0 (IBM, Armonk, NY, USA). P ≤ 0.05 was considered significant.

## Results

Forty-one knees of 41 female patients were compared with 107 knees of 104 male patients. Demographic data and sports activity level at the time of injury are given in Table 1. Male patients had significantly more strenuous sports at the time of injury (31.7% vs. 58.9%, p = 0.022).

**Table 1:** Demographics and sports activity level at the time of injury.

	**F (n = 41)**	**M (n = 107)**	**P-value**
Age	23.95±4.08	24.86±3.17	0.153
Height	162.75±5.44	175.49±6.82	0.000
Weight	63.41±11.24	76.57±10.64	0.000
BMI	23.95±4.08	24.86±3.17	0.153
Sports activity level at injury			0.022
ADL	7 (17.1%)	10 (9.3%)	
Light	3 (7.3%)	5 (4.7%)	
Moderate	18 (43.9%)	29 (27.1%)	
Strenuous	13 (31.7%)	63 (58.9%)	

BMI, body mass index; ADL, activity of daily living

The severity of osseous lesion at the lateral tibial plateau, lateral femoral condyle, medial tibial plateau, and medial femoral condyle were comparable between sexes (Table 2).

**Table 2:** Severity of osseous lesion by location.

**Severity grade**	**F (n = 41)**	**M (n = 107)**	**P-value**
Lateral tibial plateau			0.094
0	7 (17.1%)	9 (8.4%)	
1	6 (14.6%)	16 (15.0%)	
2	15 (36.6%)	26 (24.3%)	
3	13 (31.7%)	56 (52.3%)	
Lateral femoral condyle			0.113
0	16 (39.0%)	26 (24.3%)	
1	8 (19.5%)	20 (18.7%)	
2	14 (34.1%)	37 (34.6%)	
3	3 (7.3%)	24 (22.4%)	
Medial tibial plateau			0.902
0	19 (46.3%)	53 (49.5%)	
1	11 (26.8%)	24 (22.4%)	
2	8 (19.5%)	24 (22.4%)	
3	3 (7.3%)	6 (5.6%)	
Medial femoral condyle			0.134
0	37 (90.2%)	92 (86.0%)	
1	2 (4.9%)	14 (13.1%)	
2	1 (2.4%)	1 (0.9%)	
3	1 (2.4%)	0 (0.0%)	

**Fig 2:**
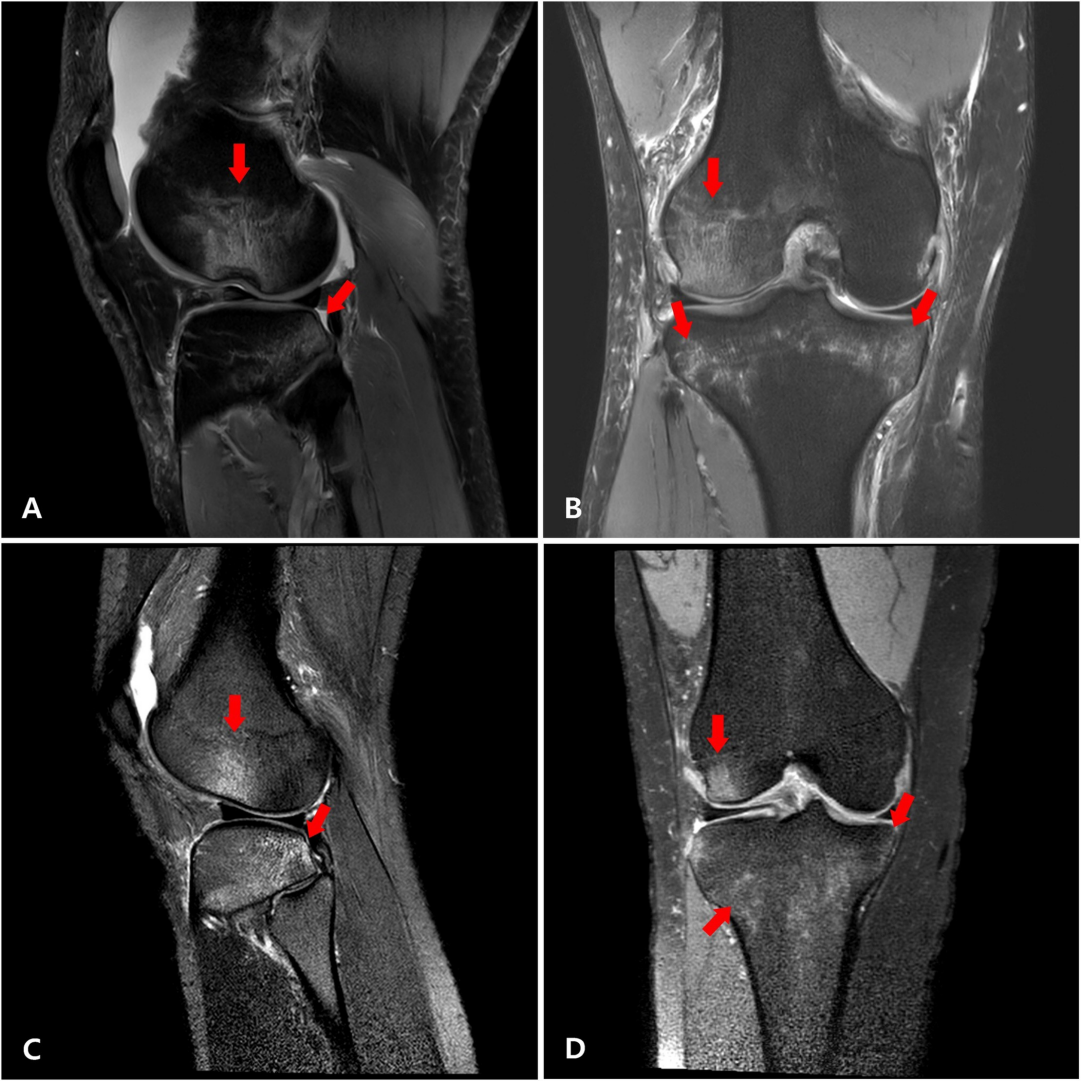
T2-weighted MRI of typical patterns of osseous lesions in acute non-contact ACL ruptures. Sagittal (A) and coronal (B) images of a male patient show osseous lesions in the central aspect of the distal lateral femoral condyle and the posterior aspect of the lateral and medial tibial plateau. Sagittal (C) and coronal (D) images of a female patient show the same pattern of osseous lesions.

Fig 2 shows the typical pattern of osseous lesions in both sexes. Grade 3 osseous lesions in the lateral tibial plateau tended to be more frequent in male patients, but there was no statistically significant difference (31.7% vs. 52.3%, p = 0.094).

Osseous lesions and meniscus tears were divided according to injury site (Table 3), and there were no significant differences between sexes in the location of tibial contusions (p = 0.21), femoral contusions (p = 0.23), or meniscus tears (p = 0.189). The most common pattern of tibial contusions in both females and males was contusions in both the medial and lateral positions (51.2%, 50.5%). The most common pattern of femoral contusions in both females and males was only lateral contusions (51.2%, 61.7%). Females tended to have more medial-only meniscal tear patterns (31.7% vs. 17.8%) and males tended to have more lateral-only meniscal tear patterns (17.1% vs. 23.4%), but there was no significant difference (p = 0.19).The compartment of injury was calculated if there was at least 1 element of osseous lesion or meniscal injury, and there were no significant differences between sexes (p = 0.20). In both sexes, injury in both compartments was the most frequent (63.4%, 57.9%). The frequency of most severe site of tibial contusion showed no difference between sexes (p = 0.71). In both sexes, the lateral compartment was the most frequent (77.1%, 72.0%).

**Table 3:** Frequency of injury site by location.

Site: Location	F (n = 41)	M (n = 107)	P-value
Tibia			0.211
Medial only	1 (2.4%)	0 (0.0%)	
Lateral only	13 (31.7%)	44 (41.1%)	
Medial and lateral	21 (51.2%)	54 (50.5%)	
Neither	6 (14.6%)	9 (8.4%)	
Femur			0.232
Medial only	0 (0.0%)	0 (0.0%)	
Lateral only	21 (51.2%)	66 (61.7%)	
Medial and lateral	4 (9.8%)	15 (14.0%)	
Neither	16 (39.0%)	26 (24.3%)	
Meniscus			0.189
Medial only	13 (31.7%)	19 (17.8%)	
Lateral only	7 (17.1%)	25 (23.4%)	
Medial and lateral	1 (2.4%)	9 (8.4%)	
Neither	20 (48.8%)	54 (50.5%)	
Compartments			0.198
Medial only	3 (7.3%)	4 (3.7%)	
Lateral only	9 (22.0%)	38 (35.5%)	
Medial and lateral	26 (63.4%)	62 (57.9%)	
Neither	3 (7.3%)	3 (2.8%)	
Most severe site of tibial contusion			
Medial = lateral	7 (20.0%)	18 (16.8%)	0.712
Medial > lateral	1 (2.9%)	2 (1.9%)	
Lateral > medial	27 (77.1%)	77 (72.0%)	
Neither	6 (14.6%)	10 (9.3%)	

Associated injuries including posterior cruciate ligament tear, medial collateral ligament tear, fibular collateral ligament tear, medial and lateral meniscus tear, and Segond fracture showed no significant difference between sexes (Table 4).

**Table 4:** Analysis of associated injuries in ligament, menisci, and Segond’s fracture.

	F (n = 41)	M (n = 107)	P-value
PCL	3 (7.3%)	13 (12.1%)	0.558
MCL	9 (22.0%)	27 (25.2%)	0.831
FCL	7 (17.1%)	18 (16.8%)	1.000
MM	14 (34.1%)	28 (26.2%)	0.415
LM	8 (19.5%)	34 (31.8%)	0.158
Segond’s fracture	1 (2.4%)	4 (3.7%)	1.000

PCL, posterior cruciate ligament; MCL, medial collateral ligament; FCL, fibular collateral ligament; MM, medial meniscus; LM, lateral meniscus

Both LTS (8.95° vs. 6.82°, p<0.0001) and MTS (8.59° vs. 6.36°, p<0.0001) were significantly larger in females, and MTD (1.80mm vs. 2.41mm, p<0.0001) was significantly shallower in females (Table 5). However, after elimination of confounding factors, multivariate logistic regression analysis (Table 6) showed that LTS and MTD were significantly different (p<0.0001), and the odds ratio to female sex was 1.464 and 0.145, respectively. MTS was not significantly different (p = 0.15).

**Table 5:** Univariate analysis of anatomical factors including tibial slope and medial tibial depth.

	**F (n = 41)**	**M (n = 107)**	**P-value**
LTS (°)	8.95±2.61	6.82±2.32	<0.0001
MTS (°)	8.59±2.95	6.36±2.66	<0.0001
MTD (mm)	1.80±0.51	2.41±0.62	<0.0001

LTS, lateral tibial slope; MTS, medial tibial slope; MTD, medial tibial depth

**Table 6:** Multivariate logistic regression analysis with variables of p<0.05 from univariate analysis.

	Odds ratio	P-value
LTS (°)	1.464 (1.217–1.761)	<0.0001
MTS (°)	1.166 (0.945–1.438)	0.153
MTD (mm)	0.145 (0.064–0.330)	<0.0001

LTS, lateral tibial slope; MTS, medial tibial slope; MTD, medial tibial depth

κ and ICC values for inter-observer and intra-observer reliability were calculated for each variable evaluated by MRI, and showed a minimum value of 0.82 indicating excellent agreement.

## Discussion

In this study, the pattern and severity of osseous lesions, meniscus and ligament ruptures between males and females did not vary significantly. Osseous lesions of the lateral tibial plateau tended to be more severe in males, but the difference was not statistically significant. In terms of anatomical factors, the females showed a greater LTS and shallower MTD, however it did not affect the sex difference in injury pattern

Fayad et al. reported a significantly higher posterolateral tibial bone contusion in females (86% vs. 61%) in 84 cases (42 males and 42 females) of ACL tear, and the maximum tibial edema depth was also greater in females (32.5mm vs. 16.0mm), likely because there is more of an effect from valgus stress in females.[22] However, there was a limitation in that the percentage of women with acute tear was significantly higher because of the lack of control over the time of MRI scans from injury. In contrast, Wittstein et al. reported that there was no significant difference in the location and severity of bone contusions and meniscal tears between sexes in non-contact ACL injuries in 73 cases (28 males and 45 females), suggesting that valgus collapse is not a more prominent mechanism of injury in females.[23] There was also a limitation in that the study only included patients under 20 years of age. Owusu-Akyaw et al. reported that there was no significant difference in the knee positions near the time of an ACL rupture with regard to knee flexion, valgus, internal tibial rotation, or anterior tibial translation between sexes in their study using MRI three-dimensional model in 30 cases (15 males and 15 females).[32]

The present study also suggests that there is no difference in non-contact injury mechanism between sexes in terms of bone bruising pattern and associated injuries in menisci and collateral ligaments. Interestingly, osseous lesions of the lateral tibial plateau tended to be more severe in males, but this difference was not statistically significant, and the reason for this could be explained with 1) higher sports activity level at the time of injury and 2) heavier weight in males than in female counterparts.

With regard to the anatomical factors, larger LTS has been reported to increase internal tibial rotation and anterior translation of the lateral tibial plateau during valgus collapse, thereby increasing the risk of ACL injury.[33, 34] On the other hand, controversy remains regarding the effect of MTS. Stijak et al.[33] reported that a larger MTS could be protective, while Hashemi et al.[12] reported that the MTS was larger in the ACL injured population. In addition, there are reports that when tibiofemoral articular congruity is low, consequent anteroposterior and rotational instability increases the risk of ACL rupture. Schneider et al. reported a significantly lower congruency of the medial and lateral compartment in females than in males in their CT study of 276 patients (138 males and 138 females) with complete ACL rupture.[35] Hashemi et al., in their MRI study, claimed that the MTD is a significant risk factor for ACL injury regardless of sex.[12] In the present study, multivariate analysis showed a greater LTS and shallower MTD in female, however it did not affect the sex difference in injury pattern. This may be attributed to the fact that this study included patients from the general population, and that the sports activity level at the time of injury was significantly higher in males.

Our study has some strengths in that it is larger than previous studies, included a general population, and included patients with MRI taken within 6 weeks from injury. The ratio of female to male participants was nearly 1 to 2.6, which leads to increased statistical power. The limitations of this study were as follows. First, it was a retrospective study and thus selection bias may have been present. Second, the sports activity level and injury mechanism at the time of injury was evaluated by chart review so in some patients, information about the specific knee position and movement was limited. Finally, study population was not limited to athletes nor limited to those injured during specific sports activity. Thus, the outcomes of our study cannot be generalized to athletes. It is necessary to conduct a comparative study by controlling the sports activity level to determine whether injury mechanism and anatomical factors differ between sexes in non-contact ACL injuries.

## Conclusion

There was no significant difference between males and females in terms of the incidence and severity of pivot shift injuries in non-contact ACL ruptures. With regard to anatomical factors, the LTS was significantly larger in females and the depth of the medial tibial plateau was significantly shallower in females.
